# Digital information-seeking behaviors among cancer survivors: associations with sociodemographic determinants, cancer history, and perceived health

**DOI:** 10.1007/s11764-025-01927-w

**Published:** 2025-11-06

**Authors:** Roberto M. Benzo, Macy K. Tetrick, Jessica L. Krok-Schoen, Theodore M. Brasky, Peter Washington, Electra D. Paskett, Frank J. Penedo, Rujul Singh, Michael Shechtman, Soledad A. Fernandez, Sachin Kumar, Mahkameh Mallahzadeh, James L. Fisher

**Affiliations:** 1 Division of Cancer Prevention and Control, Department of Internal Medicine, College of Medicine, The Ohio State University, Columbus, OH USA; 2 The Ohio State Comprehensive Cancer Center, The Ohio State University, Columbus, OH USA; 3 The Ohio State University College of Medicine, School of Health and Rehabilitation Sciences, Columbus, OH USA; 4 Division of Medical Oncology, The Ohio State University College of Medicine, Columbus, OH USA; 5 Division of Clinical Informatics and Digital Transformation, Department of Medicine, University of California , San Francisco, CA USA; 6 Departments of Psychology and Medicine, University of Miami, Miami, FL USA; 7 Sylvester Comprehensive Cancer Center, Miller School of Medicine, University of Miami, Miami, USA; 8 Department of Biomedical Informatics, College of Medicine, The Ohio State University, Columbus, OH USA; 9 Computer Science and Engineering, The Ohio State University, Columbus, OH USA; 10 Arthur G. James Cancer Hospital and Richard J. Solove Research Institute, Columbus, OH USA

**Keywords:** Cancer information, Cancer communication, Health information seeking, Cancer survivors

## Abstract

**Purpose:**

This study aimed to examine general and digital information-seeking behaviors among U.S. cancer survivors and assess how these behaviors are patterned by sociodemographic and clinical factors. The study addressed a key gap in prior research by using registry-confirmed cancer data to explore disparities in digital engagement.

**Methods:**

Data were drawn from the 2021 Health Information National Trends Survey–Surveillance, Epidemiology, and End Results Program (HINTS-SEER), which included 1,234 cancer survivors from three SEER registries. Multivariable logistic regression models evaluated associations between information-seeking (general and digital) and sociodemographic (e.g., age, sex, income) and clinical factors (e.g., cancer site, stage at diagnosis, treatment effects).

**Results:**

Most survivors (77.8%) reported having sought cancer-related information, and 73.9% used digital tools to look for health information. Survivors who were younger, female, more educated, and with higher income were significantly more likely to engage in both general and digital information-seeking when compared to older, male, or less educated. Survivors reporting cancer- and treatment-related nausea or neuropathy were more likely to engage in information-seeking when compared to survivors without these symptoms.

**Conclusions:**

Cancer survivors’ engagement in information-seeking, especially through digital tools, is shaped by heterogeneity in age, sex, education, income, and clinical experiences. These findings highlight the persistence of the digital divide in survivorship care.

Implications for Cancer Survivors.

Equity-focused efforts are needed to support digital access and literacy among underserved survivors. Tailored interventions, plain-language digital content, and provider-facilitated referrals to digital evidence-based resources may help bridge informational gaps and enhance survivorship self-management.

**Supplementary Information:**

The online version contains supplementary material available at https://doi.org/10.1007/s11764-025-01927-w.

## Background

Cancer survivorship is becoming progressively more prevalent, with over 18.6 million survivors (1 in 18 Americans) in the United States as of January 2025, a number projected to exceed 22 million by 2035 [1]. This growing incidence is attributed in part to aging populations, environmental exposures, lifestyle factors (e.g., poor dietary habits, increased alcohol and tobacco use, physical inactivity, and UV exposure), and advances in early detection, treatment efficacy, and supportive care [2–4]. Cancer survivors face complex challenges, including potentially managing multiple conditions in addition to the long-term physical and mental impacts of cancer and related treatments [5, 6]. For many cancer survivors, information-seeking is vital in care management, informed decision-making, and is linked to improved health-related quality of life (HRQoL) [6–9]. Understanding the patterns and predictors of information-seeking behaviors is critical for addressing the needs of this growing population, reducing health disparities driven by unequal access to information, and improving health outcomes.

Access to timely, accurate health information has been linked to better adherence to therapy, enhanced communication with healthcare providers, and reduced patient anxiety and distress [10–12]. For many survivors, seeking health information is an essential coping mechanism, enabling them to manage care, participate in medical decision-making, and improve their HRQoL [13–16]. Most survivors prefer comprehensive information about their illness and treatment options, positive and negative; therefore, the need for healthcare providers to facilitate patient education is apparent [8, 17]. For many survivors, understanding where to seek trustworthy cancer information is a critical component of navigating survivorship. While healthcare providers remain the most trusted source, time constraints during clinical encounters (averaging about 23 min) often limit the depth and personalization of information exchange [18, 19]. As the survivor population grows, health systems may face increasing difficulty meeting informational needs within standard visits [20]. Consequently, survivors may feel an increasing expectation to manage their own care and seek supplementary information from alternative sources (most commonly online platforms) to fill these gaps.[21]. This shift highlights the importance of understanding where survivors turn for information and how sociodemographic and clinical factors influence their choices.

Information-seeking empowers cancer survivors to engage in medical decision-making, explore treatment options, and adapt self-care strategies to their needs [6, 7, 10, 13]. Despite these benefits, unmet informational needs remain a significant issue, with up to 79% of survivors reporting difficulty obtaining the information they need [5, 22]. Previous literature has noted that barriers in information seeking include limited access to reliable sources, low health literacy, and difficulty evaluating the credibility of information [23–25]. Davis et al. used the National Cancer Institute’s Health Information National Trends Survey (HINTS) 5 (2017–2018), and found that while the majority of cancer survivors (84.7%) sought health information, only 38% used the internet for cancer-related information [15]. Their study also identified heterogeneity in information-seeking behaviors, with female sex, higher education, and higher income significantly associated with increased information-seeking, whereas lower income was linked to greater difficulty understanding health information. However, that study was limited by the composition of the HINTS sample, which historically includes a relatively small proportion (14%) of cancer survivors [26]. Comprehensive research utilizing a dataset with more robust cancer-specific attributes is needed to advance our understanding of survivors’ information-seeking behaviors.

Given the continuously evolving landscape of medical advancement and digital health resources, it is important to monitor how cancer survivors access and utilize information to ensure their needs are supported. Thus, the purpose of this study is to examine digital information-seeking behaviors and potential associations with sociodemographic and clinical factors. We are addressing a gap in the literature by leveraging the National Cancer Institute’s Health Information National Trends Survey – Surveillance, Epidemiology, and End Results Program (HINTS-SEER) of 2021 dataset, which was piloted with the goal of creating a more robust dataset of verified SEER cancer registry information, augmented with the behavioral responses of HINTS [26]. This dataset allows for a more comprehensive investigation into how a cancer diagnosis influences survivors’ information-seeking behaviors. We are particularly interested in exploring potential associations between SEER variables (e.g., cancer site, stage at diagnosis, and treatment history) and survivors’ information-seeking behaviors, as well as sociodemographic factors (e.g., age, sex, income, and education) that may influence engagement, particularly in the digital realm. Findings from this study will help inform future interventions aimed at addressing disparities in health information access and improving digital literacy among cancer survivors.

## Methods

### Survey sample and design

We used data from HINTS-SEER, which contains survey data from a sample of 1,234 U.S. cancer survivors whose diagnoses had been reported to one of three SEER Program registries in Iowa, New Mexico, and the Greater San Francisco Bay Area. The surveys were administered in 2021. Participants in the HINTS-SEER survey were cancer survivors diagnosed prior to 2018. The most recent diagnosis year available at the time of sampling was 2018. HINTS-SEER is a pilot project meant to provide more information on cancer survivors than previous iterations of HINTS; it utilized the survey format of HINTS 5, Cycle 4 (2020) with some additional survivor-specific items [27]. While HINTS surveys typically provide nationally representative data of civilian, institutionalized adult U.S. population, HINTS-SEER oversampled the cancer population through the SEER registry, allowing for data from SEER to be linked to HINTS survey responses for a more comprehensive understanding of survivors’ experiences [27]. Any additional sampling or methodological design information can be found on the HINTS website (https://hints.cancer.gov/data/survey-instruments.aspx#H2021_SM).

### Measures

## Information-seeking behavior

Information-seeking behavior was assessed using three survey questions: (1) ‘If you had a strong need to get information about cancer, where would you go first?’ with response options including *doctor or health care provider*, internet, cancer organizations, or additional sources (e.g., books, brochures, pamphlets, family, friends/co-workers, library, magazines, newspapers, or telephone information lines; see Online Resource 1)**;** (2) “Have you looked for information about cancer from any source?” (yes or no); and (3) “In the past 12 months, have you used a computer, smartphone, or other electronic means to look for health or medical information for yourself?” (yes or no). Although the HINTS-SEER survey item used the phrase “doctor or health care provider,” for consistency and to reflect the broader clinical context, this manuscript refers to this response category as “healthcare provider” throughout. This terminology encompasses the full range of professionals who may deliver survivorship information and support, including physicians, nurses, advanced practice providers, and allied health professionals.

## Sociodemographic factors

Sociodemographic factors assessed were sex as listed on the original birth certificate (female or male, as collected in HINTS-SEER), age, race/ethnicity, educational attainment, marital status, income, cancer site, and cancer stage at diagnosis. Age was categorized into three groups: 18–64, 65–74, and 75 +. Race/ethnicity responses were coded as Non-Hispanic White and Other Race/Ethnicity Groups. Educational attainment responses were high school or less, post-high school training/some college, college graduate, and post-graduate. Marital status was coded as married/living as married, divorced/separated, widowed, or single/never married. Income was categorized into three ranges: less than $50,000, $50,000 to $99,999, and $100,000 or greater. The variable for cancer stage at diagnosis was coded as localized and regional/distant. Cancer site responses were categorized as colon and rectum, female breast, prostate, or other, as defined in the SEER registry linkage. The publicly available HINTS-SEER dataset aggregates detailed SEER cancer site codes into these four categories (breast, prostate, colorectal, and other) to preserve participant confidentiality in accordance with SEER data-use policies.

Clinical variables assessed included general health, time since treatment completion, as well as cognitive impairment, neuropathy, severe fatigue, and nausea as a result of cancer treatment. Respondents self-reported general health coded as excellent, very good, good, fair, or poor. Time since last cancer treatment was coded for analysis as never had treatment, still receiving or less than 1 year, 1 to less than 5 years, 5 to less than 10 years, or 10 years or more. Cognitive impairment, neuropathy, severe fatigue, or nausea as a result of cancer treatment were all coded as yes or no.

## Statistical Analyses

Responses to the three questions were described according to categorical demographic characteristics using counts and percentages. To comply with the data use agreement, cell counts under 25 were suppressed (i.e., not shown/reported). Univariate logistic regression models were used to obtain odds ratios (OR) and 95% confidence intervals (CI) reflecting associations between having ever looked for information about cancer and having used electronic means to look for health or medical information about yourself and the following: sociodemographic factors, cancer site, stage at diagnosis, time since the completion of cancer treatment, cancer- and treatment-related effects, and perceived general health. Next, a multivariable logistic regression model was built to examine associations between these factors and the response variable information-seeking behavior (as self-reported on the HINTS-SEER survey); and a second model was fit to study the association of factors with whether subjects would use electronic means to obtain information. All statistically significant (at alpha level of 0.05) factors from the univariate models were included in the final multivariable models, with the exception of factors that were both non-significant (i.e., not significantly associated with the information-seeking behavior) and whose removal from the model had no substantive impact on other ORs in the model (resulted in at least a 15% change in at least one other OR). This approach reflects a data-driven strategy in which factors remained in the model if they were statistically significant and/or improved the overall model fit and performance. All p-values were two-sided. Statistical analyses were conducted using SPSS version 29.0.1.0 (IBM Corp. Released 2023. IBM SPSS Statistics for Windows, Version 29.0.1.0 Armonk, NY: IBM Corp).

## Results

**Fig. 1:**
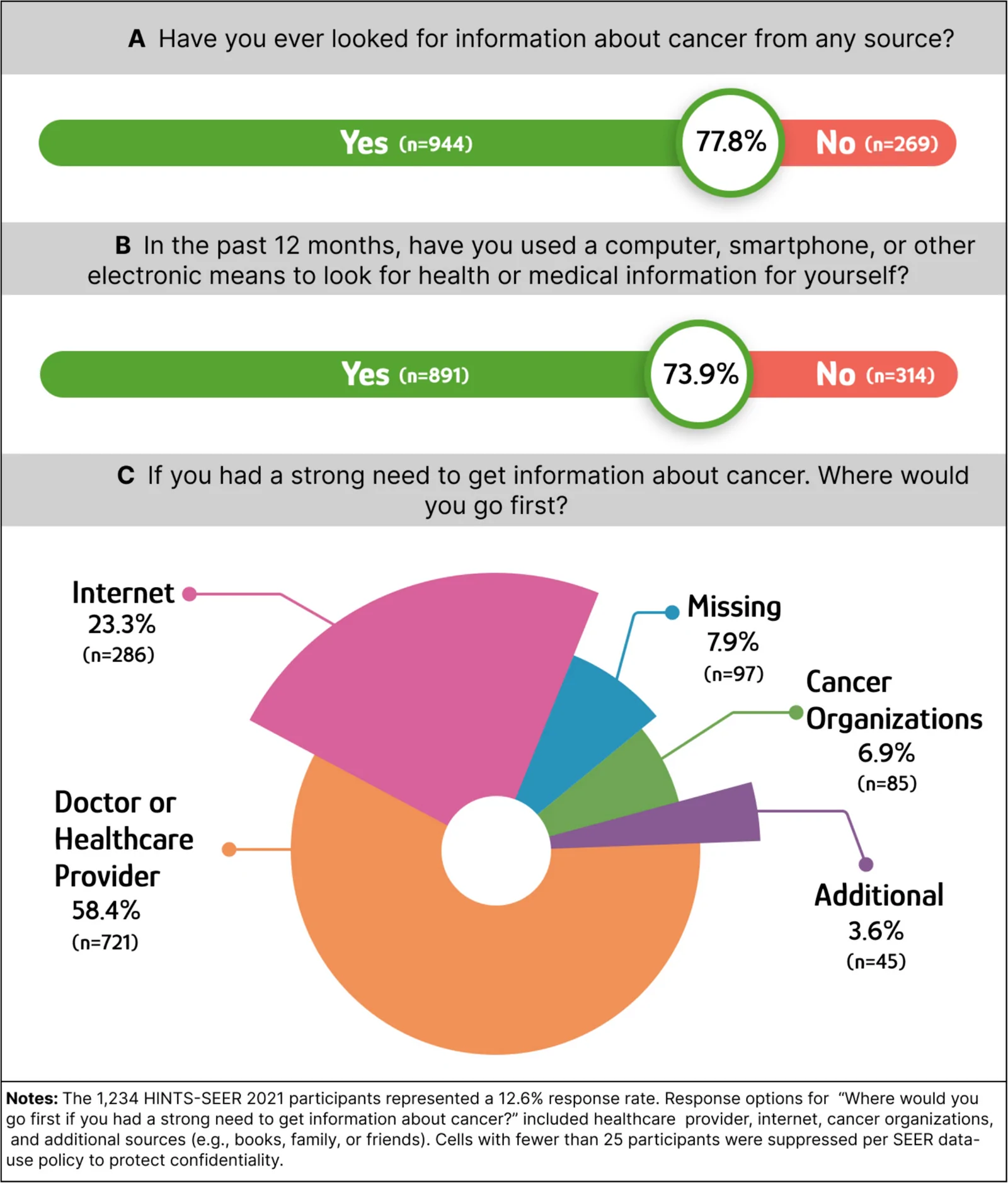
Response Rates to Information-Seeking Questions on the HINTS-SEER survey

The 1,234 participants in HINTS-SEER 2021 represent a response rate of 12.6% (a product of the proportion of consented registrants and the proportion of consenters who returned completed surveys). Figure 1 presents responses to the following survey items: (1a) “*Have you ever looked for information about cancer from any source?,*” (1b) “*In the past 12 months, have you used a computer, smartphone, or other electronic means to look for health or medical information for yourself?,*” and (1c) *“If you had a strong need to get information about cancer. Where would you go first?”.*

More than three-quarters of respondents (944 of 1,213; 77.8%) reported ever seeking cancer-related information, and nearly three-quarters (891 of 1,205; 73.9%) reported using electronic means (e.g., computer or smartphone) to look for health or medical information for themselves within the past 12 months. When asked where they would first turn for cancer-related information, most participants (58.4%) indicated a healthcare provider, followed by 23.3% who reported the internet, 6.9% who selected cancer organizations, and 3.6% who chose other sources; 7.9% of responses were missing.

Table 1 shows the responses to the questions “*Have you ever looked for information about cancer from any source?*” and “*In the past 12 months, have you used a computer, smartphone, or other electronic means to look for health or medical information for yourself?”* by demographic characteristics, cancer site, and stage at diagnosis*.*

**Table 1:** Frequencies of Responses to HINTS-SEER Questions by Personal and Clinical Data

	*Have you ever looked for information about cancer from any source?*		*In the past 12 months, have you used a computer, smartphone, or other electronic means to look for health or medical information for yourself?*	
	**Yes**	**Total**	**Yes**	**Total**
	n (%)	n	n (%)	n
Sex				
Male	406 (74.9%)	542	387 (71.4%)	542
Female	520 (81.1%)	641	487 (77.1%)	632
Age Group				
18–64	253 (88.2%)	287	252 (87.5%)	288
65–74	341 (83.2%)	410	324 (79.4%)	408
75 +	326 (67.9%)	480	293 (61.9%)	473
Race/Ethnicity				
White, Non-Hispanic	721 (80.8%)	892	683 (76.7%)	890
Other Race/Ethnicity Groups	146 (71.9%)	203	142 (71.0%)	200
Educational Attainment				
High School or Less	97 (52.2%)	186	85 (46.2%)	184
Post HS Training/Some College	238 (74.1%)	321	215 (67.6%)	318
College Graduate	273 (83.5%)	327	267 (82.4%)	324
Post-graduate	319 (91.1%)	350	308 (88.5%)	348
Marital Status				
Married/Living as Married	669 (81.1%)	825	638 (78.1%)	817
Divorced/Separated	101 (70.6%)	143	100 (69.4%)	144
Widowed	99 (68.3%)	145	76 (53.9%)	141
Single/Never Married	Suppressed	65	Suppressed	65
Income				
< $50,000	234 (63.1%)	371	217 (59.6%)	364
$50,000-$99,999	324 (82.2%)	394	300 (76.1%)	394
$100,000 +	381 (87.2%)	437	371 (85.1%)	436
Cancer Site				
Colon and Rectum	56 (68.3%)	82	53 (68.8%)	77
Female Breast	236 (83.7%)	282	212 (76.3%)	278
Prostate	213 (77.2%)	276	195 (71.2%)	274
Other	439 (76.6%)	573	431 (74.8%)	576
Stage at Diagnosis				
Localized	638 (78.0%)	818	608 (75.0%)	811
Regional/Distant	273 (78.0%)	350	254 (73.2%)	347

**Note:** Values are presented as *n (%)*, where *n* represents the number of respondents within each subgroup. Cells with fewer than 25 participants were suppressed to reduce disclosure risk per SEER data-use policy. When a suppressed value could be inferred by subtraction within a row, the next smallest cell in that row was also suppressed

Similarly, Online Resource 1, shows the responses to the question *“Where would you go first if you had a strong need to get information about cancer?”* by demographic characteristics, cancer site, and stage at diagnosis. It should be noted that a column reflecting “friends, family, books, etc.” has been suppressed due to consistent small cell sizes. As a result, cells do not sum to row totals. Responses reflecting first going to the internet were of primary importance in these analyses.

### Determinants of information seeking behaviors

As shown in the multivariable model **(see **Table 2**)**, after adjustment for confounding by other factors in the model, looking for cancer-specific information was significantly less common among males and older adults (ages 75 +). However, while marginally non-significant, individuals belonging to other race/ethnicity groups and those with regional/distant stage cancers appear to be less likely to seek information about cancer.

**Table 2:** Odds ratios and 95% confidence intervals from univariable and multivariable logistic regressions of demographic and clinical factors in association to *“Have you ever looked for information about cancer from any source?”*

	Univariable	Multivariable
	OR (95% CI)	OR (95% CI)
Sex		
Female	Ref	Ref
Male	0.70 (0.53–0.92)	0.56 (0.35–0.91)
Age Group		
18–64	Ref	Ref
65–74	0.66 (0.43–1.03)	*0.60 (0.34–1.04)*
75 +	0.28 (0.19–0.43)	0.25 (0.14–0.42)
Race/Ethnicity		
White, Non-Hispanic	Ref	Ref
Other Race/Ethnicity Groups	0.61 (0.43–0.86)	*0.68 (0.43–1.06)*
Educational Attainment		
High School or Less	Ref	Ref
Post HS Training/Some College	2.63 (1.80–3.85)	2.41 (1.45–4.00)
College Graduate	4.64 (3.08–6.99)	3.90 (2.25–6.75)
Post-graduate	9.44 (5.92–15.07)	7.28 (3.95–13.33)
Marital Status		
Married/Living as Married	Ref	
Divorced/Separated	0.56 (0.38–0.84)	
Widowed	0.50 (0.34–0.74)	
Single/Never Married	1.15 (0.59–2.24)	
Income		
< $50,000	Ref	Ref
$50,000-$99,999	2.71 (1.94–3.78)	2.32 (1.50–3.60)
$100,000 +	3.98 (2.80–5.66)	2.08 (1.29–3.35)
Cancer Site		
Colon and Rectum	Ref	Ref
Female Breast	2.38 (1.36–4.18)	2.15 (0.98–4.70)
Prostate	1.57 (0.92–2.70)	2.81 (1.30–6.05)
Others	1.52 (0.92–2.52)	1.50 (0.77–2.92)
Stage at Diagnosis		
Localized	Ref	Ref
Regional/Distant	1.00 (0.74–1.35)	0.67 (0.44–1.03)
General Health		
Excellent	Ref	
Very Good	1.09 (0.62–1.94)	
Good	0.75 (0.43–1.31)	
Fair	0.64 (0.35–1.20)	
Poor	0.72 (0.28–1.86)	
How Long Since Finished Treatment		
Never had treatment	Ref	Ref
Still Receiving or Less than 1 Year	2.53 (1.47–4.37)	3.30 (1.59–6.86)
1 to Less than 5 Years	3.22 (1.89–5.49)	3.66 (1.82–7.35)
5 to Less than 10 Years	2.32 (1.39–3.88)	2.66 (1.35–5.24)
10 Years or More	2.76 (1.72–4.45)	2.87 (1.52–5.42)
Cognitive impairment as a Result of Cancer or Cancer Treatment		
No	Ref	
Yes	2.10 (1.41–3.14)	
Neuropathy as a Result of Cancer or Cancer Treatment		
No	Ref	Ref
Yes	1.46 (1.05–2.04)	1.60 (1.01–2.55)
Severe Fatigue as a Result of Cancer or Cancer Treatment		
No	Ref	
Yes	1.49 (1.10–2.03)	
Nausea as a Result of Cancer or Cancer Treatment		
No	Ref	Ref
Yes	2.37 (1.56–3.59)	1.59 (0.93–2.73)
Year of Diagnosis (continuous, per year)	1.00 (0.99–1.02)	

Conversely, looking for cancer-specific information was significantly more common with increasing educational attainment, those with higher incomes, and those with a history of cancer treatment, regardless of the time since the treatment was received. Also, those who experienced neuropathy as a result of cancer or treatments were more likely than those who had not to seek cancer information.

As shown in the multivariable model **(*see***Table 3**)**, after adjustment for confounding by other factors in the model, using electronic means to look for health or medical information about oneself was significantly less common among males, those ages 75 and older, widowed, and with regional/distant stage cancer. Using electronic means to look for health or medical information about oneself was significantly more common among those with higher educational attainment, higher incomes ($100,000 +), and nausea as a result of cancer or cancer treatment.

**Table 3:** Odds ratios and 95% confidence intervals from univariable and multivariable logistic regressions of demographic and clinical factors in association to *“In the past 12 months, have you used a computer, smartphone, or other electronic means to look for health or medical information for yourself?”*

	Univariable	Multivariable
	OR (95% CI)	OR (95% CI)
Sex		
Female	Ref	Ref
Male	0.74 (0.57–0.97)	0.57 (0.39–0.81)
Age Group		
18–64	Ref	Ref
65–74	0.55 (0.36–0.84)	0.68 (0.40–1.16)
75 +	0.23 (0.16–0.35)	0.27 (0.16–0.45)
Race/Ethnicity		
White, Non-Hispanic	Ref	
Other Race/Ethnicity Groups	0.74 (0.53–1.05)	
Educational Attainment		
High School or Less	Ref	Ref
Post HS Training/Some College	2.43 (1.67–3.53)	1.74 (1.07–2.83)
College Graduate	5.46 (3.63–8.20)	3.42 (2.01–5.82)
Post-graduate	8.97 (5.78–13.91)	7.20 (4.01–12.94)
Marital Status		
Married/Living as Married	Ref	Ref
Divorced/Separated	0.64 (0.43–0.94)	0.71 (0.43–1.19)
Widowed	0.33 (0.23–0.48)	0.47 (0.28–0.79)
Single/Never Married	1.24 (0.65–2.37)	1.52 (0.63–3.65)
Income		
< $50,000	Ref	Ref
$50,000-$99,999	2.16 (1.58–2.95)	1.45 (0.96–2.21)
$100,000 +	3.87 (2.76–5.41)	1.76 (1.09–2.87)
Cancer Site		
Colon and Rectum	Ref	
Female Breast	1.46 (0.83–2.54)	
Prostate	1.12 (0.65–1.93)	
Others	1.35 (0.80–2.26)	
Stage at Diagnosis		
Localized	Ref	Ref
Regional/Distant	0.91 (0.69–1.21)	0.63 (0.43–0.92)
General Health		
Excellent	Ref	
Very Good	1.06 (0.61–1.84)	
Good	0.71 (0.41–1.20)	
Fair	0.47 (0.26–0.84)	
Poor	1.43 (0.49–4.18)	
How Long Since Finished Treatment		
Never had treatment	Ref	
Still Receiving or Less than 1 Year	1.38 (0.80–2.38)	
1 to Less than 5 Years	1.78 (1.05–3.03)	
5 to Less than 10 Years	1.44 (0.85–2.42)	
10 Years or More	1.60 (0.98–2.60)	
Cognitive impairment as a Result of Cancer or Cancer Treatment		
No	Ref	
Yes	1.75 (1.22–2.51)	
Neuropathy as a Result of Cancer or Cancer Treatment		
No	Ref	
Yes	1.41 (1.03–1.92)	
Severe Fatigue as a Result of Cancer or Cancer Treatment		
No	Ref	
Yes	1.39 (1.04–1.86)	
Nausea as a Result of Cancer or Cancer Treatment		
No	Ref	Ref
Yes	1.97 (1.36–2.86)	2.24 (1.35–3.70)
Year of Diagnosis (continuous, per year)	1.01 (1.00–1.03)	

## Discussion

In this study of U.S. cancer survivors using HINTS-SEER 2021 data, we found that survivors’ sociodemographic and clinical characteristics strongly influenced both general and digital cancer information-seeking behaviors. Overall, 78% of participants had sought cancer-related information from any source, and 74% reported using digital tools (e.g., computers or smartphones) for health or medical information. Information-seeking was significantly more common among younger, female, higher-educated, and higher-income survivors, and less common among those who were older, widowed, or diagnosed with advanced-stage disease. Survivors reporting cancer- or treatment-related symptoms such as nausea or neuropathy were also more likely to seek information, suggesting that ongoing symptom burden may motivate information-seeking during survivorship.

These findings align with and extend prior national research. Davis et al. [15] found that 89% of survivors sought cancer-related information from any source and that 37% used the Internet specifically to look for cancer-related information. The similarity in “first source” patterns (22% of survivors in Davis et al. and 23% in our study identifying the Internet as their initial source) supports the stability of these trends across time. The apparent differences between studies reflect variation in question framing: our study assessed the use of any digital device to obtain health or medical information, while Davis et al. focused solely on cancer-related Internet use. Together, these data indicate that most survivors actively seek information but vary in how they do so, reflecting a persistent digital divide influenced by age, education, income, and health status.

We observed that younger age, female sex, higher educational attainment, and greater household income were strongly associated with seeking cancer information. Survivors who were widowed, older (especially age 75 +), or male were significantly less likely to seek information. These findings are consistent with previous research by Rutten et al. (2016), who documented similar patterns over a decade of HINTS data, showing that older age, lower education, and lower income were consistently linked to lower tendency for information-seeking behaviors from any source [7]. Our study builds on that work by evaluating patterns among cancer survivors included in three SEER cancer registries and identifying that widowed status and advanced-stage diagnosis were additional barriers to information-seeking in survivorship.

Among cancer survivors, digital information-seeking was significantly more common among those with higher educational attainment and income. Survivors with a post-graduate education were over seven times as likely (OR: 7.20, 95% CI: 4.01–12.94) to use electronic tools for health information than those with a high school education or less. Likewise, those with higher income levels demonstrated significantly greater odds of digital engagement. These findings align with Melhem et al. (2023), who found that higher education, income, and employment status independently predicted digital literacy and eHealth engagement among cancer survivors [28]. Our results reinforce that educational and financial resources are enablers of digital information access, contributing to the digital divide (i.e., the gap between individuals or groups who have access to and can effectively use digital technologies and those who cannot).

Conversely, older adults and widowed survivors were significantly less likely to use digital tools to access cancer-related information. This is consistent with both Rutten et al. and Melhem et al., who reported that age and social support influence access to digital resources and confidence and motivation to use them effectively [7, 28]. The digital divide thus reflects not only differences in technology access, but also in skills, trust, and usage patterns among survivors. Conversely, Davis et al. reported time since diagnosis as the only significant predictor of internet use for cancer-related information. This discrepancy may reflect the differences in datasets: HINTS-SEER links HINTS survey responses with clinical characteristics provided by central cancer registries in SEER, in contrast to Davis et al., who relied on self-reported cancer history, potentially underestimating clinical predictors.

Survivors diagnosed with regional or distant stage disease were significantly less likely to use electronic means to seek health information. This finding aligns with prior research showing that poorer health status, greater symptom burden, and advanced cancer stage are linked to lower engagement in online or general information-seeking behaviors [10, 11, 29]. Individuals with advanced disease often experience physical and cognitive fatigue, anxiety, or treatment intensity that limit their motivation or ability to search for information [29]. The abundance of online health information can also be overwhelming or distressing, further discouraging active seeking. Additionally, selection bias cannot be ruled out, as survivors with advanced disease may have been underrepresented in HINTS-SEER due to illness severity or non-response to mailed surveys. Together, these findings underscore the need for tailored, low-burden communication approaches and proactive clinician engagement to ensure that information needs are met even among those with poorer health or advanced disease.

Beyond sociodemographic patterns, we found that survivors who reported treatment-related nausea and neuropathy were more likely to seek cancer-related information. This suggests that survivors managing ongoing or late-emerging symptoms may turn to external sources to support symptom self-management. Jenkins et al. (2001) highlighted that most survivors, regardless of age, desire comprehensive information about side effects and symptom management [17], reflecting a persistent demand for detailed guidance throughout the cancer care continuum. Rutten et al. (2016) similarly reported that information-seeking among survivors often centers on managing long-term treatment effects, especially in the absence of regular provider contact [7]. The differing symptom associations across models (neuropathy for general information-seeking and nausea for digital information-seeking) may reflect small subgroup sample sizes and wide confidence intervals rather than meaningful behavioral differences.

A key finding of this study is the contrast between survivors who first turned to a health care provider’ versus the internet as their first source for cancer-related information. Overall, 58.4% of survivors identified their health care provider as their first source of cancer information, whereas 23.3% reported the internet as their primary source. As shown in Online Resource 1, a higher proportion of older, less educated, and lower-income survivors identified their health care provider as their initial source, whereas relatively higher proportions of younger, higher-educated, and higher-income survivors reported using the internet as their first source. These results align with national trends, showing that although trust in healthcare professionals remains high, survivors’ reliance on healthcare providers versus online sources varies with age, education, income, and digital literacy, patterns also linked to differences in access and confidence [7, 21]. Given the complexity of cancer care and related treatment information, as well as the rapid evolution of digital technology that allows survivors to search and access vast amounts of information, limited clinical time may further encourage survivors’ use of online resources to supplement, rather than replace, communication with healthcare providers. Strengthening provider-initiated referrals to credible, evidence-based digital content could help balance the personalization of clinical guidance with the scalability of digital education, thereby improving patient activation and informed self-management. Similarly, Davis et al. (2021) found that many survivors continue to seek cancer-related information well into survivorship to address unresolved questions and evolving health concerns [15]. Melhem et al. (2023) emphasized that effective digital engagement depends heavily on survivors’ digital literacy and access[28], factors that influence their ability to manage symptom burden through independent information-seeking.

Despite these high rates of information-seeking and growing use of digital tools, a disconnect remains between survivors’ preferred and available information sources. While most survivors in our sample identified their healthcare provider as the first source they would turn to for cancer-related information, the increasing reliance on online tools reflects both necessity and opportunity. Rutten et al. (2016) reported that while healthcare providers remained the most frequently used and trusted first source of cancer information (rising from 19.6% in 2003 to 44.4% in 2008), use of the internet remained relatively stable, reflecting the enduring and evolving dual role of clinicians and digital platforms in survivorship communication. However, not all survivors are equally equipped to navigate digital tools, particularly those facing literacy, access, or confidence barriers; highlighting the need for equity-focused strategies that ensure all survivors can obtain reliable information in formats they trust.

Taken together, our findings support a growing body of literature indicating that information-seeking among cancer survivors is shaped by a complex interplay of sociodemographic and clinical factors. Survivors with fewer resources (i.e., economic, educational, or social) face systemic barriers to accessing information critical to informed decision-making. Addressing these gaps will require proactive, equity-centered approaches that extend digital literacy support, offer plain-language resources, and bridge the divide between clinical and digital care channels.

### Implications for survivors and practice

Our findings underscore the importance of understanding why certain subgroups, particularly survivors with advanced-stage disease, lower income, or limited digital access, are less likely to seek health information online. Beyond barriers of access, factors such as perceived reliability of online content, trust in healthcare providers, and emotional or cognitive burden may strongly influence engagement [29–31]. These findings suggest that low information-seeking among these groups may reflect not only resource limitations but also psychological and informational fatigue. Large-scale reviews have noted that few digital health interventions are designed for older adults or advanced-stage patients, populations facing the greatest unmet informational needs [32]. Pilot psychoeducational programs like ENCOURAGE demonstrated selective benefits among distressed survivors but limited overall efficacy [33], reinforcing that such digital approaches remain exploratory. Likewise, early eHealth systems such as CHESS illustrated the potential of technology to enhance information access and support [34], but modern interventions must now address contemporary barriers of trust, reliability, and digital literacy. Moving forward, mixed-methods and longitudinal research should clarify the cognitive, emotional, and contextual factors that influence survivors’ engagement with digital health information, strengthening the evidence base needed to develop accessible, evidence-informed digital communication strategies that enhance survivorship care across diverse populations. Insights from such work can guide the creation of adaptive, theory-driven digital interventions that evolve with survivors’ changing informational needs and bridge the gap between clinical communication and self-directed information-seeking.

### Strengths and limitations

This study’s use of HINTS-SEER 2021 data enabled a more clinically precise understanding of survivors’ information behaviors by linking cancer characteristics included in SEER (e.g., site, stage at diagnosis, treatment history) with behavioral survey data. Compared to previous HINTS datasets, this provides a stronger foundation for understanding how clinical and sociodemographic variables interact to shape health information-seeking patterns.

This study is limited by its cross-sectional design, preventing causal inference. The relatively low sample size (N = 1,234) and response rate (12.6%) may also introduce selection bias, potentially overrepresenting more engaged or health-literate survivors. Underserved cancer survivors may be underrepresented in this sample to the extent that underserved survivors may be less likely to have participated; as a result, the findings reported here may not be generalizable to all cancer survivors. Further, the survey’s mailed paper-based format may have under-sampled survivors who face literacy or accessibility barriers. Additionally, the ‘Other’ cancer site category encompasses a heterogeneous mix of cancers (e.g., lung, melanoma, hematologic, gynecologic) because SEER-linked site codes were aggregated to minimize disclosure risk in the publicly available dataset. This restriction limited our ability to analyze site-specific variations in information-seeking behaviors. Finally, while our data provide valuable insight into who is and isn’t seeking information, we did not assess the quality, effectiveness, or comprehension of the information obtained, which are critical factors highlighted by Davis et al. [15].

## Conclusion

Our findings highlight persistent differences in cancer-related and digital information-seeking behaviors among U.S. cancer survivors**.** Survivors who were older, male, widowed, or diagnosed with advanced cancer stages were less likely to engage in these behaviors, placing them at potential risk for unmet informational and subsequent clinical needs. While healthcare providers remain the most trusted and frequently used source of cancer information, many survivors also turn to digital platforms to fill informational gaps between clinical encounters [7, 21]. Recognizing the complementary roles of providers and digital resources is essential for creating equitable and integrated survivorship communication strategies.

These findings underscore the importance of improving the representativeness of future national surveys to capture survivors with lower digital access or poorer health status and of examining how trust and perceived reliability shape engagement with online health information. Future research should prioritize mixed-methods and longitudinal designs to better understand these dynamics and inform the development of evidence-based digital communication strategies that meet survivors’ needs across sociodemographic and clinical contexts. In parallel, translational efforts should focus on developing and testing tailored digital health interventions (e.g., animations, gamified modules, and provider-guided referrals) that enhance digital literacy, improve confidence in online resources, and ensure all survivors are equipped to manage their health post-treatment. This approach aligns with the HINTS program’s goal of advancing equitable, data-driven cancer communication and informing interventions that bridge the gap between clinical and digital care.

## Supplementary Information

Below is the link to the electronic supplementary material.

ESM 1 (DOCX 18.9 KB)

## Data Availability

The datasets analyzed during this study are available from the corresponding author on reasonable request. This study used data from the 2021 Health Information National Trends Survey–Surveillance, Epidemiology, and End Results Program (HINTS-SEER) database. Any additional sampling or methodological design information can be found on the HINTS website (https://hints.cancer.gov/data/survey-instruments.aspx#H2021_SM).
