# Supplementary material for: Digital information-seeking behaviors among cancer survivors: associations with sociodemographic determinants, cancer history, and perceived health
Source: J Cancer Surviv. 2025 Nov 6;20(5):2233–44. doi: 10.1007/s11764-025-01927-w (PMC13723649; doi:10.1007/s11764-025-01927-w)
Supplement: Supplementary file 1 [file 11764_2025_1927_MOESM1_ESM.docx]

| **Online Resource 1. Frequencies of responses to the question “Where would you go first if you had a strong need to get information about cancer?” according to demographic characteristics, cancer site, and stage at diagnosis among HINTS-SEER participants** | | | | | | |
| --- | --- | --- | --- | --- | --- | --- |
|  | Healthcare Provider | Internet | Cancer Organizations | Additional | Missing | Total^1^ |
|  | N (%) | N (%) | N (%) | N (%) | N (%) | N |
| Sex |  |  |  |  |  |  |
| Male | 313 (56.2%) | 137 (24.6%) | Suppressed | Suppressed | 46 (8.3%) | 557 |
| Female | 390 (60.5%) | 144 (22.3%) | Suppressed | Suppressed | 46 (7.1%) | 645 |
| Age Group |  |  |  |  |  |  |
| 18-64 | 147 (50.7%) | 93 (32.1%) | Suppressed | Suppressed | Suppressed | 290 |
| 65-74 | 235 (56.6%) | 113 (27.2%) | Suppressed | Suppressed | Suppressed | 415 |
| 75+ | 318 (64.8%) | 74 (15.1%) | 38 (7.7%) | Suppressed | Suppressed | 491 |
| Race/Ethnicity |  |  |  |  |  |  |
| White, Non-Hispanic | 538 (59.4%) | 220 (24.3%) | Suppressed | Suppressed | Suppressed | 906 |
| Other Race/Ethnicity Groups | 109 (53.4%) | 47 (23.0%) | Suppressed | Suppressed | Suppressed | 204 |
| Educational Attainment |  |  |  |  |  |  |
| High School or Less | 118 (62.8%) | Suppressed | Suppressed | Suppressed | Suppressed | 188 |
| Post HS Training/Some College | 207 (62.9%) | 71 (21.6%) | Suppressed | Suppressed | Suppressed | 329 |
| College Graduate | 180 (54.2%) | 87 (26.2%) | Suppressed | Suppressed | Suppressed | 332 |
| Post-graduate | 197 (55.8%) | 103 (29.2%) | Suppressed | Suppressed | Suppressed | 353 |
| Marital Status |  |  |  |  |  |  |
| Married/Living as Married | 483 (57.8%) | 211 (25.2%) | 55 (6.6%) | Suppressed | Suppressed | 836 |
| Divorced/Separated | 80 (54.1%) | 33 (22.3%) | Suppressed | Suppressed | Suppressed | 148 |
| Widowed | 100 (68.0%) | Suppressed | Suppressed | Suppressed | Suppressed | 147 |
| Single/Never Married | 39 (60.0%) | Suppressed | Suppressed | Suppressed | Suppressed | 65 |
| Income |  |  |  |  |  |  |
| <$50,000 | 230 (61.2%) | 61 (16.2%) | 37 (9.8%) | Suppressed | Suppressed | 376 |
| $50,000-$99,999 | 245 (60.8%) | 97 (24.1%) | Suppressed | Suppressed | Suppressed | 403 |
| $100,000+ | 238 (53.6%) | 127 (28.6%) | Suppressed | Suppressed | Suppressed | 444 |
| Cancer Site |  |  |  |  |  |  |
| Colon and Rectum | 49 (59.8%) | Suppressed | Suppressed | Suppressed | Suppressed | 82 |
| Female Breast | 176 (62.0%) | 58 (20.4%) | Suppressed | Suppressed | Suppressed | 284 |
| Prostate | 164 (57.5%) | 63 (22.1%) | Suppressed | Suppressed | Suppressed | 285 |
| Others | 332 (56.9%) | 141 (24.2%) | 46 (7.9%) | Suppressed | Suppressed | 583 |
| Stage at Diagnosis |  |  |  |  |  |  |
| Localized | 488 (58.6%) | 195 (23.4%) | 54 (6.5%) | Suppressed | Suppressed | 833 |
| Regional/Distant | 199 (56.2%) | 85 (24.0%) | 31 (8.8%) | Suppressed | Suppressed | 354 |
| **Note**: Cells with fewer than 25 participants were suppressed to decrease disclosure risk. In addition, when cell sizes could be determined by subtraction within the row, the cell with next smallest size was also suppressed. ‘Doctor’ reflects the exact wording used in the HINTS-SEER survey item (‘Where would you go first if you had a strong need to get information about cancer?’) | | | | | | |
